# Early changes in plasma DNA levels of mutant KRAS as a sensitive marker of response to chemotherapy in pancreatic cancer

**DOI:** 10.1038/s41598-017-08297-z

**Published:** 2017-08-11

**Authors:** Marzia Del Re, Caterina Vivaldi, Eleonora Rofi, Enrico Vasile, Mario Miccoli, Chiara Caparello, Paolo Davide d’Arienzo, Lorenzo Fornaro, Alfredo Falcone, Romano Danesi

**Affiliations:** 1 Unit of Clinical Pharmacology and Pharmacogenetics, Department of Clinical and Experimental Medicine, University of Pisa, Pisa, Italy; 2 Medical Oncology Unit, Department of Translational Research and New Technologies in Medicine, University of Pisa, Pisa, Italy; 3 Department of Clinical and Experimental Medicine, University of Pisa, Pisa, Italy; 4 Sant’Anna School of Advanced Studies, Department of Medical Sciences, Pisa, Italy

## Abstract

Pancreatic cancer (PDAC) is still lacking of reliable markers to monitor tumor response. CA 19-9 is the only biomarker approved, despite it has several limitations in sensitivity and specificity. Since mutations of KRAS occur in more than 90% of tumors, its detection in circulating free tumor DNA (cftDNA) could represent a biomarker to monitor chemotherapy response. Twenty-seven advanced PDAC patients given first-line 5-fluorouracil, irinotecan and oxaliplatin or gemcitabine and nab-paclitaxel were enrolled. Three ml of plasma were collected: 1) before starting chemotherapy (baseline); 2) at day 15 of treatment; and 3) at each clinical follow-up. cftDNA was extracted and analysed for KRAS mutations (^mut^KRAS) by digital droplet PCR. Nineteen patients displayed a ^mut^KRAS in baseline plasma samples. There was a statistically significant difference in progression-free survival (PFS) and overall survival (OS) in patients with increase *vs*. stability/reduction of cftDNA in the sample collected at day 15 (median PFS 2.5 vs 7.5 months, p = 0.03; median OS 6.5 vs 11.5 months, p = 0.009). The results of this study demonstrate that cftDNA ^mut^KRAS changes are associated with tumor response to chemotherapy and support the evidence that ^mut^KRAS in plasma may be used as a new marker for monitoring treatment outcome and disease progression in PDAC.

## Introduction

Pancreatic adenocarcinoma (PDAC) represents the fourth leading cause of cancer-related deaths in Western countries and only 10–20% of patients are diagnosed with a resectable disease^1^. Chemotherapy represents the milestone of treatment of advanced disease; indeed, among the combinations developed in recent years, 5-fluorouracil, irinotecan and oxaliplatin (FOLFIRINOX), and the association of gemcitabine and nab-paclitaxel (GEMnPAC) have demonstrated improved survival and represent now the standard of care in first-line treatment^2, 3^.

However, since pancreatic tumor tissue is surrounded by a dense fibrotic stroma, the evaluation of tumor response to therapy is particularly challenging and imaging techniques do not always provide an accurate estimate, despite the significant costs^4, 5^. For this reason, the monitoring of these tumors by blood markers is a valuable alternative and is based on the evaluation of the Carbohydrate Antigen 19-9 (CA 19-9) levels, the standard serum marker for pancreatic cancer. Although CA 19-9 has been approved to monitor tumor response, it has several limitations including: (1) low sensitivity and specificity (estimated to be around 79% and 82%, respectively); (2) occurrence of unspecific changes in serum samples; (3) poor accuracy in the identification of small tumors^6, 7^. These issues limit the utility of CA 19-9 as a biomarker and highlight the need for new biomarkers to complement the imaging in order to obtain a more effective monitoring of these patients and improve the clinical outcome. Alternative approaches for PDAC monitoring are represented by circulating tumor cells (CTCs), which may predict poor disease outcome^8^, though their use is limited by false negative tests and high costs.

The majority of pancreatic cancers (from 75 to 95%) harbor a KRAS mutation at codon 12^9^ and a significant difference in survival was observed in patients with detectable or undetectable KRAS mutations (^mut^KRAS) in circulating free tumor DNA (cftDNA)^10^. Moreover, the presence of ^mut^KRAS in plasma samples after surgical resection of PDAC has been associated with poor survival^11, 12^. In addition, looking at the specific type of mutation, the presence of KRAS p.G12V seems to be associated with a decrease in survival of pancreatic cancer patients^13^.

Tissue availability is recognized as a crucial issue, as there are several limitations to obtain repeated biopsies (i.e. invasiveness) and to capture tumor heterogeneity due to the practical constraints to collect multiple samples. In contrast, the detection of somatic mutations in cftDNA released in plasma from apoptotic and necrotic tumor cells both from primary tumor and metastatic lesions is feasible and affordable in terms of costs^14^. Thus, cftDNA may reflect the tumor burden in cancer patients and is a valuable option for a better monitoring of response; it may complement imaging in order to obtain a more effective management of patients, identify early resistance and improve clinical outcome^15^.

Therefore, the aim of the present study was to monitor the allelic burden of ^mut^KRAS in patients with advanced PDAC undergoing first-line combination chemotherapy. In particular, the correlation between early variations of cftDNA ^mut^KRAS after 15 days of treatment with response to therapy was explored, in order to provide an effective tool to predict the effectiveness of therapy early after its beginning.

## Results

### Study population

A total of 27 patients with locally advanced (n = 4; 15%) and metastatic (n = 23; 85%) PDAC were included in this biomarker study (Table 1). Median age was 68 years (range 49–77).

**Table 1:** Characteristics of patients.

Total	N (%)
	27 (100%)
**Gender**	
Male	14 (52%)
Female	13 (48%)
**Age**	
Median (range)	68 (49–77)
**ECOG Performance Status**	
0	23 (85%)
1	4 (15%)
**Chemotherapy regimen**	
FOLFIRINOX	13 (52%)
GEMnPAC	14 (48%)
**Stage**	
III	4 (15%)
IV	23 (85%)
**Primary tumor location**	
Head	13 (48%)
Body-tail	14 (52%)
**Baseline CA 19**-**9**	
Normal (0–37 U/ml)	7 (26%)
Abnormal (<59 ULN)	11 (41%)
Abnormal (≥59 ULN)	9 (33%)
**Baseline KRAS cftDNA status**	
Wild type	8 (29.6%)
^mut^KRAS	19 (70.4%)
**KRAS mutations**	
p.G12D	14 (73%)
p.G12R	2 (11%)
p.G12V	2 (11%)
p.G13D	1 (5%)
**Median baseline** ^**mut**^ **KRAS (copies/ml, range)**	2100 (80–64800)

At a median follow up of 7.6 months, 13 patients (54%) underwent disease progression and 6 (22%) died. Median progression-free survival (PFS) and overall survival (OS) were 7.4 and 11.5 months, respectively. Response rate was 38% and disease control rate (DCR) defined as the sum of partial response (PR) and stable disease (SD) was 73%.

### Analysis of ^mut^KRAS cftDNA at baseline

At baseline, 19 out of 27 patients (70.4%) were carriers of one ^mut^KRAS allele; 14 subjects were carriers of p.G12D, while 2 patients had the p.G12V, 2 ﻿subjects﻿ the p.G12R and another the p.G13D. In ^mut^KRAS patients, the amount of cftDNA ranged from 80 to 64800 copies/ml (Table 1). Eight patients were negative for cftDNA ^mut^KRAS at baseline (3 patients were stage III and 5 were stage IV); one of them turned to positivity at day 15 and another one at the first radiological evaluation (2 months after treatment start). There was no statistically significant difference in ^mut^KRAS cftDNA positivity at baseline in patients with metastatic *vs*. locally advanced disease (p = 0.065). Besides, there was no statistically significant difference in ^mut^KRAS positivity by stratifying patients by gender (p = 0.103), age (p = 0.087), performance status (PS) (p = 1), disease site (p = 0.103), and stage (p = 0.065).

No statistically significant differences in median PFS and OS in patients with baseline positive or negative cftDNA ^mut^KRAS were found (PFS: 7.4 months *vs*. not reached, p = 0.24; OS: 11.5 months *vs*. not reached, p = 0.16; Table 2).

**Table 2:** Univariate analysis for the correlation of PFS and OS with clinical status of patients.

	PFS		OS	
	Months (median)	*p*	Months (median)	*p*
**Age**				
<Median *vs*. ≥Median	7.4 *vs*. 8.5	0.38	NR *vs*.11.5	0.42
**Gender**				
Male *vs*. Female	7.5 *vs*. 7.3	0.65	11.5 *vs*. NR	0.35
**Stage**				
III *vs*. IV	7.4 *vs*. 7.3	0.36	NR *vs*. 11.5	0.4
**ECOG Performance Status**				
0 *vs*. 1	7.5 *vs*. 2.8	0.32	11.5 *vs*. 8.5	0.06
**Primary tumor location**				
Head *vs* Body-tail	7.4 *vs*. 7.5	0.92	NR *vs*. 11.5	0.55
**Baseline CA 19**-**9**				
Normal *vs*. Abnormal (<59 ULN *vs*. Abnormal (≥59 ULN)	NR *vs*. 7.3 *vs*. 2.8	0.06	NR *vs*. NR *vs*. 11.5	0.28
**Liver metastases**				
Yes *vs*. No	7.2 *vs*. 8.5	0.37	11.5 *vs*. NR	0.51
**Chemotherapy regimen**				
FOLFIRINOX *vs*. GEMnPAC	7.4 *vs*. 7.5	0.99	NR *vs*. 11.5	0.4
**cftDNA detectable**				
Yes *vs*. No	7.4 *vs*. NR	0.24	11.5 *vs*. NR	0.16
**Early** ^**mut**^ **KRAS cftDNA variation**				
Increase *vs*. No increase	2.5 *vs*. 7.5	**0.03**	6.5 *vs*. 11.5	**0.009**

ULN: upper limit of normality.

On the contrary, there was a statistically significant difference between cftDNA ^mut^KRAS positivity at baseline and the presence of liver metastasis (p = 0.008) or abnormal CA 19-9 levels (p = 0.011).

### Monitoring ^mut^KRAS cftDNA during treatment and correlation with outcome

Twenty-five out of 27 patients (17 positive cftDNA ^mut^KRAS and 8 wild type) had more than one blood sample drawn. One patient died within a month of study entry and another one withdrew the consent to participate in the study.

**Figure 1:**
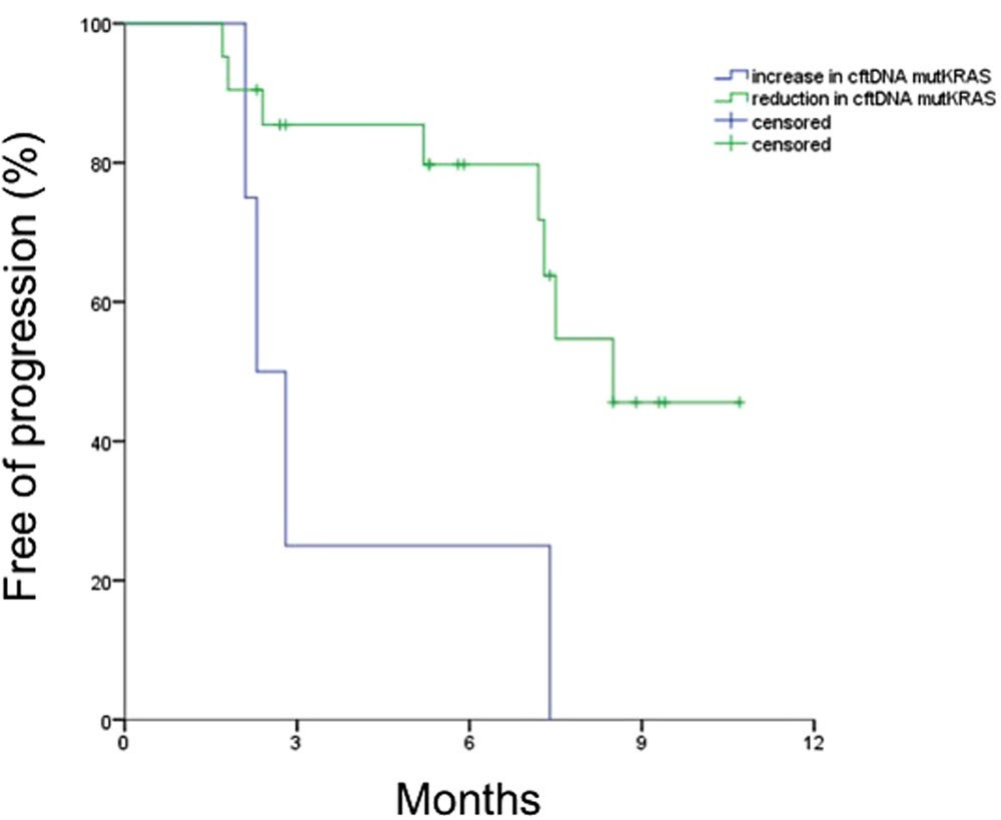
PFS according to early ^mut^KRAS cftDNA variation (increase vs. reduction).

There was a statistically significant difference in PFS between patients displaying an increase *vs*. stability/reduction of ^mut^KRAS cftDNA at the 15 day-sample (median PFS 2.5 *vs*. 7.5 months, p = 0.03, Fig. 1). In particular, according to the radiological evaluation 2 months after the beginning of treatment, all patients displaying cftDNA increase at the 15th day (3 baseline ^mut^KRAS and 1 wild type that became ^mut^KRAS) had disease progression.

Interestingly, a statistically significant difference was also observed in median OS (6.5 *vs*. 11.5 months, p = 0.009) in patients displaying an increase *vs*. reduction of ^mut^KRAS cftDNA, respectively. None of the other parameters (sex, age, stage, PS, primary tumor location, baseline CA 19-9) was significantly correlated with PFS and/or OS (Table 2).

**Figure 2:**
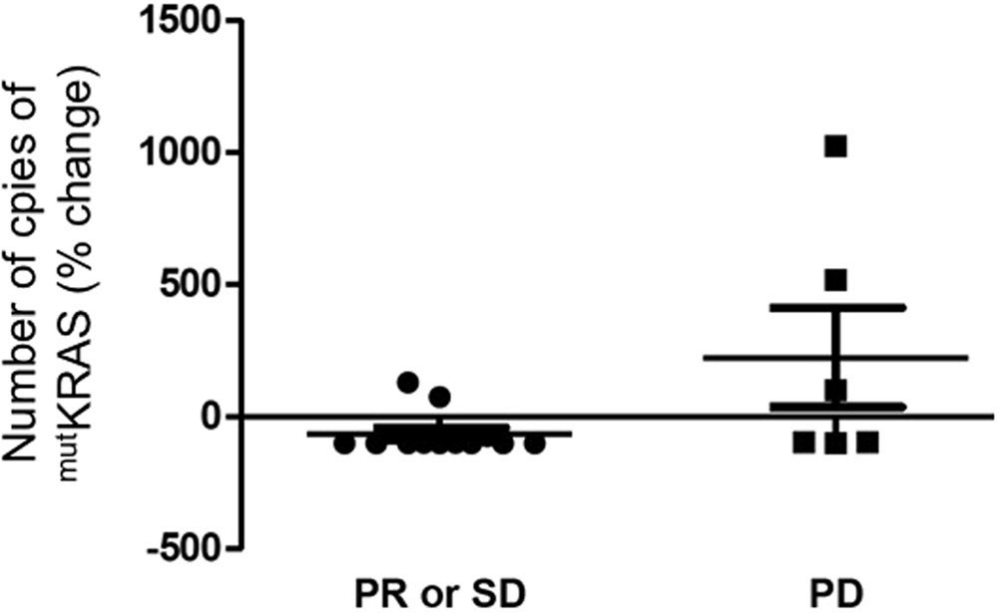
Plot showing early ^mut^KRAS cftDNA variations (% change in number of copies) in patients with PR or SD *vs*. PD.

The early ^mut^KRAS cftDNA variation did not correlate with tumor response (Fisher’s exact test p = 0.09; Mann-Whitney test p = 0.156) even if a trend toward better DCR in patients with early ^mut^KRAS cftDNA decrease was found (Fisher’s exact test p = 0.08; Mann-Whitney test p = 0.059, Fig. 2).

**Figure 3:**
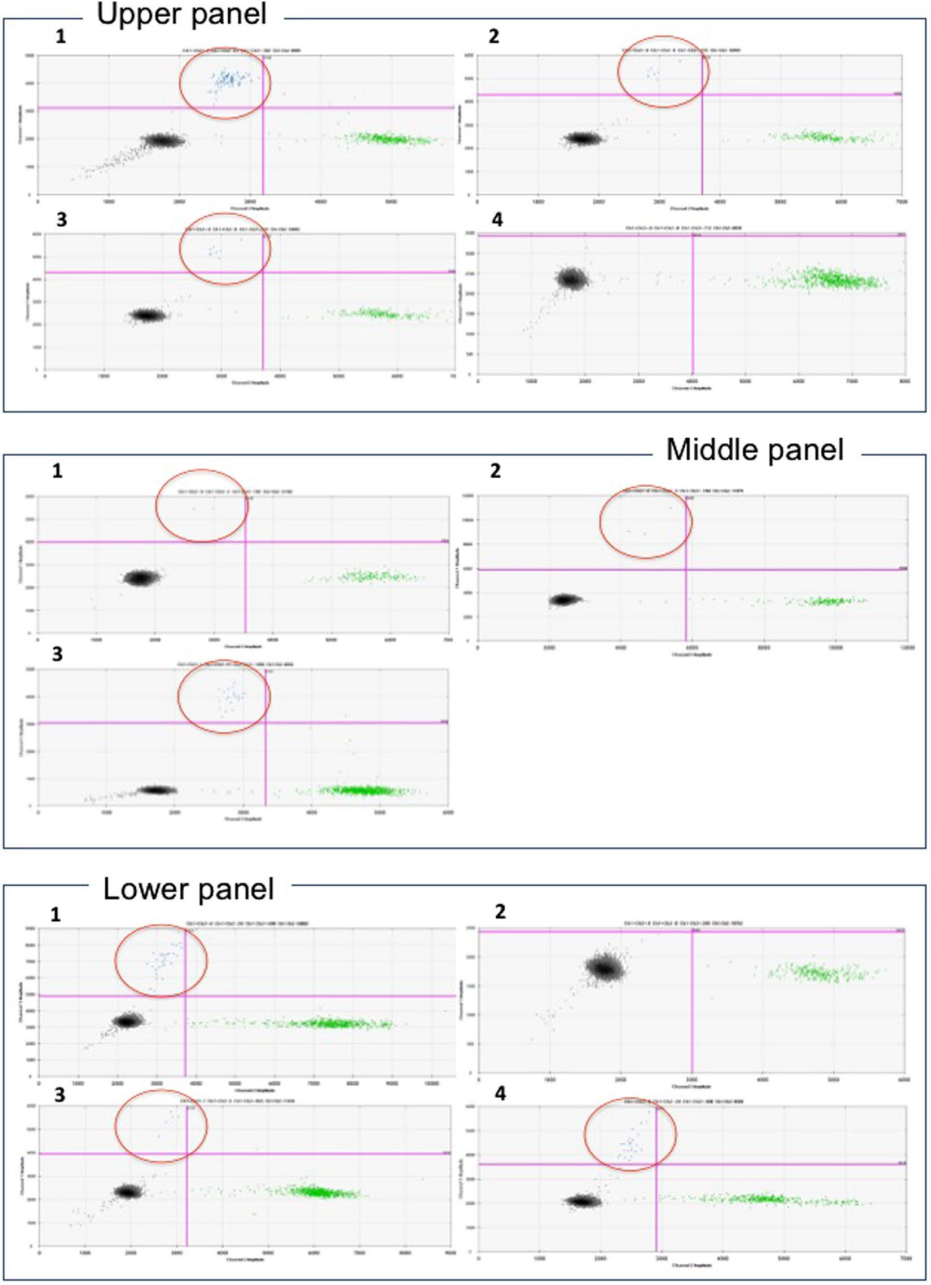
Representative ddPCR plots showing ^mut^KRAS cftDNA variations during follow up of patients. The ^mut^KRAS cftDNA are the blue dots circled in red. *Upper panel*: metastatic patient who developed PR to first-line FOLFIRINOX. (1) Baseline detection of KRAS p.G12D (11600 copies/ml); (2) p.G12D declined after 15 days (900 copies/ml); (3) p.G12D still reduced after 2 months (130 copies/ml); (4) pG12D is undetectable after 7 months of chemotherapy (0 copies/ml). *Middle panel*: patient with local recurrence who developed early PD during first-line FOLFIRINOX. (1) Baseline detection of KRAS p.G12D (240 copies/ml); (2) slight increase of p.G12D (245 copies/ml) after 15 days; (3) p.G12D increase (2700 copies/ml) after 2 months. This patient died due to PD two weeks after radiological re-evaluation at 2 months. *Lower panel*: metastatic patient who developed PR and subsequent early PD to GEMnPAC. (1) Baseline detection of KRAS p.G12D (3200 copies/ml); (2) undetectable cftDNA at day 15 (0 copies/ml); (3) increase of p.G12D (900 copies/ml) after 2 months; (4) further increase of p.G12D (2800 copies/ml) corresponding to radiologically-confirmed PD at 4 months. Clinical condition progressively deteriorated and patients died because of PD 5 months after diagnosis.

**Figure 4:**
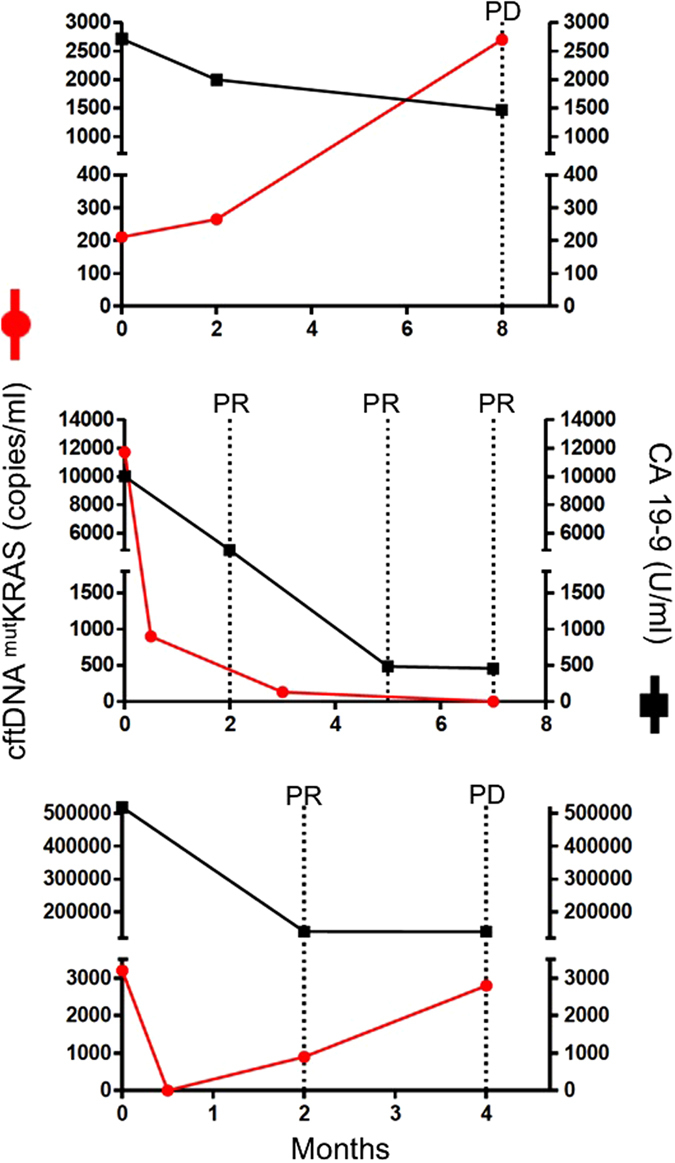
Changes in cftDNA ^mut^KRAS levels compred to CA 19-9 in a case of early PD (upper panel), PR (middle panel) and early PR followed by PD (lower panel).

Noteworthy, the comparison of ^mut^KRAS cftDNA changes in samples taken at baseline and after 2 months (at the time of first radiological evaluation) demonstrated that median PFS was 7.5 months for patients whose cftDNA ^mut^KRAS decreased vs 2.8 months of subjects with increasing cftDNA ^mut^KRAS levels (p = 0.028). Figure 3 reports the results of 3 representative patients as an example of PR (Fig. 3a), early PD (Fig. 3b) and early disease response followed by progression (Fig. 3c), while on the other hand, Fig. 4 reports the same patients comparing the cftDNA ^mut^KRAS to the CA 19-9 monitoring.

## Discussion

The present study provides the evidence that the change of ^mut^KRAS cftDNA levels between baseline *vs*. day 15 is an early predictor of clinical outcome. To the best of our knowledge, the present study shows for the first time that cftDNA ^mut^KRAS variation is an early marker of response to treatment in PDAC. In our pilot study, all patients having an early increase in cftDNA ^mut^KRAS underwent rapid disease progression, supporting the hypothesis that cftDNA ^mut^KRAS changes are associated with tumor dynamics. Experimental evidences demonstrate that cftDNA is a valuable biomarker to predict the response or resistance to treatment not only with targeted therapies, but also with chemotherapy^16^.

While previous studies demonstrated that pancreatic cancer patients with detectable ^mut^KRAS cftDNA before or after initiation of chemotherapy have a shorter survival time^13, 17, 18^, our results did not find a statistically significant difference in median PFS and OS in patients with baseline positive or negative ^mut^KRAS cftDNA (median PFS 7.4 months *vs*. not reached, p = 0.24; median OS 11.5 months *vs*. not reached, p = 0.16, respectively). Despite enrolling also patients with locally advanced disease, we did not find any difference between ^mut^KRAS cftDNA positivity in locally advanced *vs*. metastatic patients. The lack of difference in OS between ^mut^KRAS cftDNA positive and negative patients could be explained by the relatively low number of survival events - at the time of analysis 22% of the patients were deceased - and by differences in the cftDNA ^mut^KRAS detection method used in our study. Of note, the technique used in the present study is characterized by a high sensitivity, as it is able to detect small amounts of cftDNA (median baseline KRAS copies/ml: 2100; range: 80–64800 copies/ml). In fact, in the present study, 70.4% of patients had detectable ^mut^KRAS cftDNA prior to treatment, whereas in previously published studies detectability ranged from 0% to 62.2%^13, 17–20^. This high positivity rate may be dependent on the sensitivity of the method used for the analysis being digital droplet PCR (ddPCR) the most sensitive compared to other techniques.

Two out of 6 ^mut^KRAS cftDNA negative patients became positive during treatment, one after 15 days and another after 8 weeks, respectively. It is unclear if this is simply due to one or more of the following reasons: (1) increase in tumor burden; (2) molecular heterogeneity within the primary tumor; (3) changes in dynamics of ^mut^KRAS cftDNA released from cells; or (4) the emergence of metastases or CTCs with different molecular clones contributing to the cftDNA pool released. It would be extremely interesting, in the future, to analyze tumor biopsies from these patients to confirm the proportion of ^mut^KRAS.

The present study was aimed to explore whether early changes in cftDNA levels might be useful to monitor treatment response. We observed a trend towards better DCR in patients with early ^mut^KRAS cftDNA decrease during treatment. Recently, an article on this topic has been published^10^, demonstrating that changes in cftDNA levels were related to the radiological response. However, our study has been conducted on a larger and more homogeneous cohort of patients, treated with improved first-line combination regimens, and investigated the role of early variations ^mut^KRAS cftDNA, at variance with other publications in the field.

Under this point of view, future studies should focus on this issue also by comparing paired tumor biopsies and plasma from these patients, to confirm the correlation between ^mut^KRAS cftDNA and treatment outcome. Indeed, the use of cftDNA approach and of a sensitive method, such as ddPCR, allow the detection of very small amounts of mutated clones in a large background of wild type DNA, minimizing the false negative data. In this context, monitoring the amount or the appearance of tumor molecular alterations (i.e. KRAS or other mutations like TP53, SMAD4) in cftDNA during treatment is a promising and reliable tool to monitor treatment resistance or response, as it reflects the tumor dynamics and tissue heterogeneity. In conclusion, although the present study examines a small cohort of patients, the results are in support of the hypothesis that cftDNA may be used as a new marker to monitor treatment outcome and disease progression in PDAC potentially earlier than radiological findings, suggesting that cftDNA ^mut^KRAS changes are associated with tumor dynamics.

## Patients and Methods

### Patients

The study enrolled 27 consecutive patients with histologically proven advanced PDAC undergoing first-line chemotherapy with FOLFIRINOX or GEMnPAC as per standard practice. Patients were accrued from November 2015 to December 2016 at the Medical Oncology Unit of the University Hospital of Pisa. Treatment was administered until PD, unacceptable toxicity or patient refusal. Patients underwent standard disease evaluation by imaging according to RECIST 1.1 criteria: computed tomography (CT) of abdomen and chest was performed at baseline and every 8 weeks until PD. Disease control rate (DCR) was estimated as the percentage of patients who have achieved PR and SD during treatment. CA 19-9 was determined at every radiological evaluation. Data on PS, adverse events, serum chemistry, hematology and concomitant medications were collected at every chemotherapy cycle in a prospective database. Blood samples were drawn by venous puncture as follows: (1) baseline (immediately before chemotherapy administration); (2) after 15 days (before the second cycle of FOLFIRINOX or on day 15 of cycle 1 of GEMnPAC); and (3) at first radiological evaluation.

The study was approved by the Ethics Committee of Pisa University Hospital and conducted in accordance to the principles of the Declaration of Helsinki; all patients gave their signed informed consent before blood collection and cftDNA analysis.

### Plasma collection, cftDNA extraction and KRAS analysis

Six ml of blood were collected in tubes containing EDTA and centrifuged at 3000 rpm for 10 min at 4 °C within 2 hours after blood drawing; plasma samples were stored at −80 °C until analysis. cftDNA was extracted using a QIAamp Circulating nucleic acid kit (Qiagen^®^, Valencia, CA, USA) from 3 ml of plasma following the manufacturer’s protocol and cftDNA was eluted with 50 μl of elution buffer.

The analysis was performed with a ddPCR KRAS Screening Multiplex Kit (BioRad®, Hercules, CA) and the results were confirmed using single mutation assays (BioRad®, Hercules, CA) to detect p.G12D, p.G12V, p.G12R, p.G13D. Based on the information reported in the technical annex, the MiQE Context Sequence is ATTATTTTTATTATAAGGCCTGCTGAAAATGACTGAATATAAACTTGTGGTAGTTGGAGCT[G/C]GTGGCGTAGGCAAGAGTGCCTTGACGATACAGCTAATTCAGAATCATTTTGTGGACGAATA (Entrez Gene ID 3845, RefSeq accessions NM_004985, NM_033360; Ensemble Accessions ENST00000311936, ENST00000256078, ENST00000556131, ENST00000557334).

Briefly, PCR reactions were assembled as *per* manufacturer instruction and the amplification protocol was standardized for all mutations to the following conditions: 95 °C × 10 min, 94 °C × 30 s and 55 °C × 60 s (35 cycles), 98 °C × 10 min, and 4 °C hold. As a positive control for ^mut^KRAS, the cftDNA from 30 patients with known ^mut^KRAS colorectal cancer was used, while the DNA extracted from plasma of 43 healthy blood donors was used as negative control. The droplet-reader was used for fluorescence signal quantification; the QuantaSoft (BioRad®, Hercules, CA) software measured the number of positive vs. negative droplets for both fluorophores (FAM/HEX) and their ratio was fitted to a Poisson distribution to determine the copy number/ml of the target molecule in the input reaction. A fluorescence intensity threshold of 3000 was set as a cut-off point and all droplets above this threshold were scored as positive for ^mut^KRAS. The sample was considered as KRAS positive when at least 3 positive HEX droplets were identified above the threshold level.

### Statistical analyses

OS was measured from the date of the first cycle of chemotherapy to death or last follow-up visit. PFS was measured from the date of the first cycle of chemotherapy to disease progression or death, whichever occurred first. PFS and OS were estimated using the Kaplan-Meier product-limit method. Shapiro-Wilk test was performed to verify normality of distributions of cftDNA ^mut^KRAS variations; the latter were correlated with tumor response by Mann-Whitney test and Fisher’s exact test and with OS and PFS by log-rank test. Significance was set at p < 0.05 for two-tailed tests. Characteristics of patients, including and KRAS status and CA 19-9, were compared by the Fisher’s exact test. Univariate analysis was performed to correlate PFS and OS with demographics and clinical status of patients, i.e., ECOG PS, stage of disease, primary tumor location, chemotherapy regimen, site of disease, liver metastases, CA 19-9 levels and ^mut^KRAS. Cut-off data of the analyses was January 2017. The cftDNA ^mut^KRAS variation was evaluated both as a categorical variable (increase *vs*. no increase) and as a continuous variable considered as the percentage decreasing with respect to the baseline amount. Statistical analyses were carried out using the statistical software package SPSS 19.0 (SPSS, Chicago, IL, USA).
